# Utility of Ki-67 index combined with alpha-fetoprotein and lactate dehydrogenase in distinguishing mature from immature ovarian teratomas in children

**DOI:** 10.3389/fneur.2026.1757328

**Published:** 2026-04-21

**Authors:** Xiazhe Li, Zhiyong Zhong, Yanwei Qi, Yingxin Gong

**Affiliations:** Second Department of General Surgery, Hebei Children’s Hospital, Hebei Provincial Clinical Research Center for Child Health and Disease, Shijiazhuang, Hebei, China

**Keywords:** alpha-fetoprotein, biomarkers, immature teratoma, Ki-67, lactate dehydrogenase, ovarian teratoma, pediatrics, prognostic factors

## Abstract

**Background:**

Accurate differentiation between mature and immature ovarian teratomas in children remains a diagnostic challenge. Current histopathological grading is subjective, and reliable preoperative biomarkers are lacking. This study aimed to evaluate the diagnostic and prognostic value of Ki-67, alpha-fetoprotein (AFP), and lactate dehydrogenase (LDH), individually and in combination.

**Methods:**

We retrospectively analyzed 82 pediatric patients (≤18 years) with histologically confirmed ovarian teratomas, including 45 mature and 37 immature cases. Serum AFP and LDH levels and Ki-67 proliferation indices were compared between groups. Multivariate logistic regression and receiver operating characteristic (ROC) analyses were performed to identify independent predictors and assess diagnostic performance. Recurrence-free survival (RFS) was evaluated using Kaplan–Meier and Cox regression analyses.

**Results:**

AFP, LDH, and Ki-67 levels were significantly higher in the immature group than in the mature group (all *p* < 0.001). Ki-67 expression increased progressively with higher pathological grades (*P* for trend < 0.001). In multivariate analysis, Ki-67 > 30% (OR 7.16, 95% CI 3.09–16.58), AFP > 500 ng/mL (OR 5.42, 95% CI 2.31–12.75), and LDH > 300 U/L (OR 3.04, 95% CI 1.32–6.98) were independent predictors of immaturity. The combined model (AFP + LDH + Ki-67) achieved the highest diagnostic accuracy (AUC = 0.96), outperforming any single marker (*p* < 0.05). During a median follow-up of 38 months, recurrence occurred in 8 patients (10.3%), all with immature teratomas. High Ki-67 expression (>30%) was independently associated with shorter RFS (HR = 4.62, 95% CI 1.48–14.46, *p* = 0.009).

**Conclusion:**

The combined assessment of Ki-67, AFP, and LDH provides a robust biomarker panel for differentiating immature from mature ovarian teratomas in children and predicting recurrence risk. Integration of proliferative and metabolic indicators into pathological evaluation may enhance diagnostic precision and support individualized management in pediatric ovarian germ cell tumors.

## Introduction

Ovarian teratoma represents one of the most common germ cell tumors in the pediatric population, accounting for approximately 30–40% of all ovarian neoplasms in children (1, 2). Histologically, these tumors are classified into mature and immature types according to the degree of tissue differentiation (3, 4). While mature teratomas are benign and usually have an excellent prognosis, immature teratomas exhibit malignant potential and are characterized by a higher risk of recurrence and metastasis, particularly in advanced pathological grades (2, 5–7). The accurate distinction between mature and immature ovarian teratomas remains a major clinical challenge (8). Histopathologic grading, primarily based on the Norris classification, is currently the diagnostic gold standard (9–12). However, this approach is inherently subjective and prone to inter-observer variability, especially in borderline or heterogeneous lesions (3, 12). Moreover, reliable non-invasive preoperative biomarkers are still lacking, making it difficult to predict tumor behavior or guide individualized surgical and follow-up strategies in pediatric patients (13).

Serum biomarkers play an important role in the diagnosis and monitoring of germ cell tumors. Alpha-fetoprotein (AFP) is widely used as a tumor marker reflecting embryonic differentiation, and markedly elevated levels have been reported in immature ovarian teratomas and other malignant germ cell tumors (14–17). Lactate dehydrogenase (LDH) serves as an indicator of cellular metabolism and tumor burden, and has been associated with disease aggressiveness and poor prognosis in various malignancies (18, 19). In addition, Ki-67, a classical proliferation marker expressed during all active phases of the cell cycle, provides a quantitative index of tumor growth activity and has been correlated with histological grade and recurrence risk in several ovarian neoplasms (20–22). Despite these insights, current research remains limited by several gaps.

The malignant potential of ovarian teratomas is closely linked to cellular proliferation, differentiation, and metabolic activity (23–26). Ki-67, a nuclear protein expressed during active phases of the cell cycle, reflects proliferative activity and correlates positively with tumor grade and histologic immaturity (20, 21, 27, 28). AFP, produced by embryonic hepatocytes and germ cells, indicates aberrant differentiation and persistence of embryonic features (14, 15). LDH reflects enhanced glycolytic metabolism and tissue hypoxia, both characteristic of aggressive tumor behavior (18, 19). Together, these markers represent complementary dimensions of proliferation, metabolism, and differentiation, forming a composite “biological malignancy spectrum” that may better capture the heterogeneity of immature ovarian teratomas. Moreover, few investigations have systematically evaluated the combined diagnostic and prognostic performance of AFP, LDH, and Ki-67 in differentiating mature from immature ovarian teratomas.

Based on these considerations, this study aimed to clarify the diagnostic and prognostic significance of Ki-67, AFP, and LDH in pediatric ovarian teratomas. By comparing their expression levels between mature and immature subtypes and assessing their independent associations with pathological grade and recurrence, we sought to establish a combined biomarker model that integrates proliferative, metabolic, and differentiation indices. Such an approach may provide a more objective and quantitative supplement to conventional histopathological grading, thereby improving preoperative risk stratification and long-term management in children with ovarian teratomas.

## Methods

### Study design and population

This study was designed as a retrospective case–control analysis conducted at Hebei Children’s Hospital. Pediatric patients aged ≤18 years who were pathologically diagnosed with ovarian teratomas between January 2015 and December 2023 were included. A total of 82 patients met the inclusion criteria, comprising 45 cases of mature teratoma and 37 cases of immature teratoma confirmed by postoperative histopathology.

All clinical, laboratory, and pathological data were obtained from the hospital’s electronic medical record system. The study protocol was reviewed and approved by the Institutional Ethics Committee of Hebei Children’s Hospital. Given its retrospective design, the requirement for individual informed consent was waived, and all procedures complied with the principles of the Declaration of Helsinki.

### Inclusion and exclusion criteria

Patients were enrolled according to the following inclusion and exclusion criteria: (1) Pathologically confirmed diagnosis of ovarian teratoma, including both mature and immature types, based on postoperative histopathological evaluation. (2) Availability of preoperative serum tests for AFP and LDH performed within 2 weeks prior to surgery. (3) Presence of Ki-67 immunohistochemical (IHC) staining data obtained from formalin-fixed paraffin-embedded tumor specimens. (4) Complete clinical records, including demographic information, tumor characteristics, surgical details, and follow-up outcomes. (5) Age ≤18 years at the time of diagnosis.

Exclusion criteria: (1) Presence of other concurrent malignant tumors or mixed ovarian germ cell neoplasms (e.g., teratoma combined with yolk sac tumor, dysgerminoma, or embryonal carcinoma). (2) Patients who had received preoperative chemotherapy or radiotherapy, or had incomplete treatment history. (3) Cases with insufficient clinical, imaging, or pathological data, or poor-quality IHC slides precluding reliable Ki-67 evaluation. (4) Patients lost to follow-up or with unclear recurrence/survival information.

### Pathological diagnosis and grading

The pathological diagnosis of ovarian teratomas was established based on postoperative histological evaluation in accordance with the Norris grading system and the 2020 WHO Classification of Female Genital Tumours (9, 10). Immature teratomas were graded as Grade 1–3 according to the quantity of immature neuroepithelial tissue observed under low-power fields (×40): Grade 1, rare immature neural foci (<1 per low-power field); Grade 2, moderate immature neuroepithelium (1–3 foci per low-power field); and Grade 3, abundant immature tissue (≥4 foci per low-power field). The degree of immaturity was determined by both the quantity and the distribution of primitive neural elements.

For analytical purposes, patients were classified into two groups—mature teratoma and immature teratoma—for intergroup comparison of clinicopathological and biomarker characteristics. All histopathological slides were independently re-evaluated by two experienced pathologists who were blinded to all clinical and laboratory information. Any discrepancies in grading or interpretation were resolved through joint review and consensus discussion to ensure diagnostic consistency.

### Biomarker measurement

#### Serum AFP and LDH assays

Peripheral venous blood samples were collected from all patients before surgical intervention. Serum alpha-fetoprotein (AFP) concentrations were measured using a chemiluminescent immunoassay on an automated analyzer (reference range: <20 ng/mL). Serum lactate dehydrogenase (LDH) levels were determined by the enzymatic rate method (reference range: 120–250 U/L). All biochemical assays were conducted in the hospital’s central clinical laboratory under standardized internal and external quality-control procedures to ensure measurement consistency.

#### Ki-67 immunohistochemistry evaluation

For Ki-67 immunohistochemical (IHC) staining, formalin-fixed paraffin-embedded (FFPE) tumor tissues were sectioned at 4 μm and stained using a monoclonal anti–Ki-67 antibody (clone MIB-1, Dako, Denmark) following the manufacturer’s protocol. At least 1,000 tumor cell nuclei were counted under high-power fields, and the Ki-67 labeling index was calculated as the percentage of positively stained nuclei among total counted tumor cells. Based on prior reports and internal distribution characteristics, Ki-67 ≤ 30% was defined as *low expression*, and >30% as *high expression*. All slides were independently reviewed by two pathologists blinded to the patients’ clinical and laboratory data; discrepancies were resolved by consensus discussion.

### Follow-up and outcome assessment

All patients were regularly followed up through outpatient visits and telephone interviews. Follow-up evaluations included physical examination, serum AFP and LDH monitoring, and abdominal or pelvic ultrasonography or MRI every 3–6 months during the first 2 years and annually thereafter. The follow-up period was calculated from the date of surgery to the date of the last contact or recurrence. The median follow-up duration for the entire cohort was 38 months (interquartile range: 26–54 months). Recurrence was defined as the radiological or histological reappearance of teratomatous lesions confirmed by imaging or pathology after complete surgical resection. Recurrence-free survival (RFS) was defined as the time interval between the date of surgery and the date of the first recurrence. Patients without recurrence at the last follow-up were censored at that time point. All survival data were verified independently by two investigators based on clinical records and imaging reports.

### Statistical analysis

All statistical analyses were performed using SPSS version 26.0 (IBM Corp., United States), R version 4.3.0 (R Foundation for Statistical Computing, Austria), and GraphPad Prism 9.0 (GraphPad Software, United States). Continuous variables were tested for normality using the *Shapiro–Wilk test*. Normally distributed data were expressed as mean ± standard deviation (SD) and compared using the *independent-samples t-test*, whereas non-normally distributed data were expressed as median [interquartile range, IQR] and analyzed using the *Mann–Whitney U test*. Categorical variables were presented as counts and percentages and compared using the *χ^2^ test* or *Fisher’s exact test*, as appropriate.

A multivariate linear regression model was further applied to assess the independent relationships of AFP and LDH with Ki-67, after adjusting for potential confounders such as age, tumor size, and pathological grade. Univariate and multivariate logistic regression analyses were conducted to identify predictors of immature teratoma occurrence. Variables with *p* < 0.10 in univariate analysis were included in the multivariate model, and results were presented as odds ratios (ORs) with 95% confidence intervals (CIs).

For diagnostic performance, receiver operating characteristic (ROC) curves were plotted for individual biomarkers (AFP, LDH, Ki-67) and the combined logistic model. The area under the curve (AUC), sensitivity, specificity, and Youden index were calculated, and DeLong’s test was used to compare AUCs between models. For prognostic analyses, Kaplan–Meier survival curves were generated to evaluate recurrence-free survival (RFS) according to biomarker expression, and log-rank tests were used for group comparisons. Independent prognostic factors were identified using the Cox proportional hazards regression model, with results reported as hazard ratios (HRs) and 95% CIs. All tests were two-tailed, and *p* < 0.05 was considered statistically significant.

## Results

### Baseline characteristics

A total of 82 pediatric patients diagnosed with ovarian teratomas were included, comprising 45 cases of mature teratomas and 37 cases of immature teratomas. The baseline characteristics of the two groups are summarized in Table 1. The mean age did not differ significantly between the groups (9.82 ± 2.94 years vs. 10.36 ± 3.08 years, *p* = 0.357). In contrast, the mean tumor size was markedly larger in patients with immature teratomas than in those with mature teratomas (8.62 ± 2.77 cm vs. 5.18 ± 2.06 cm, *p* < 0.001). The laterality distribution (left vs. right ovary) was comparable between the two groups (*p* = 0.824). Serum biomarker analysis showed significantly higher levels of alpha-fetoprotein (AFP) and lactate dehydrogenase (LDH) in the immature group (median 428.50 [183.00–708.00] ng/mL and 364.15 ± 97.86 U/L, respectively) compared with the mature group (median 12.40 [6.70–21.10] ng/mL and 211.38 ± 64.72 U/L; both *p* < 0.001). Similarly, the Ki-67 proliferation index was markedly elevated in immature teratomas (35.17 ± 12.06%) compared with mature lesions (8.42 ± 5.58%; *p* < 0.001). Within the immature teratoma group, 12 (32.4%) were classified as Grade 1, 15 (40.5%) as Grade 2, and 10 (27.0%) as Grade 3, according to the Norris grading system.

**Table 1:** Baseline characteristics of children with mature and immature ovarian teratomas.

Variable	Mature teratoma (*n* = 45)	Immature teratoma (*n* = 37)	*p*-value
Age (years, mean ± SD)	9.82 ± 2.94	10.36 ± 3.08	0.357
Tumor size (cm, mean ± SD)	5.18 ± 2.06	8.62 ± 2.77	<0.001
Laterality, *n* (%)			
Left ovary	23 (51.11%)	18 (48.65%)	0.824
Right ovary	22 (48.89%)	19 (51.35%)	
Serum AFP (ng/mL, median [IQR])	12.40 [6.70–21.10]	428.50 [183.00–708.00]	<0.001
Serum LDH (U/L, mean ± SD)	211.38 ± 64.72	364.15 ± 97.86	<0.001
Ki-67 index (% positive cells, mean ± SD)	8.42 ± 5.58	35.17 ± 12.06	<0.001
Pathological grade (within immature group), n (%)	–		
G1	–	12 (32.43%)	–
G2	–	15 (40.54%)	–
G3	–	10 (27.03%)	–

Data are presented as mean ± SD or median [IQR] as appropriate.

Comparisons between mature and immature groups were conducted using independent-samples *t*-test or *χ*^2^ test as applicable.

*P* < 0.05 was considered statistically significant.

AFP, alpha-fetoprotein; LDH, lactate dehydrogenase.

### Expression of Ki-67 index

The Ki-67 proliferation index differed significantly between mature and immature ovarian teratomas (Table 2). The mean Ki-67 index in the immature group was 35.17 ± 12.06%, which was markedly higher than that in the mature group (8.42 ± 5.58%, *p* < 0.001). Within the immature teratoma subgroup, a graded increase in Ki-67 expression was observed with advancing histologic grade according to the Norris classification: 21.30 ± 7.60% in Grade 1, 34.80 ± 9.50% in Grade 2, and 47.50 ± 11.20% in Grade 3. A significant linear trend was confirmed (*P* for trend < 0.001), suggesting that Ki-67 expression correlates positively with the degree of tissue immaturity.

**Table 2:** Ki-67 expression in different pathological subtypes of ovarian teratomas.

Group/grade	*n*	Ki-67 index (% positive cells, mean ± SD)	*P*-value
Mature teratoma	45	8.42 ± 5.58	
Immature teratoma (total)	37	35.17 ± 12.06	<0.001
Grade 1 (G1)	12	21.30 ± 7.60	
Grade 2 (G2)	15	34.80 ± 9.50	
Grade 3 (G3)	10	47.50 ± 11.20	
*P* for trend			<0.001

Ki-67 index represents the percentage of positively stained tumor nuclei determined from pathology reports.

The index was significantly higher in immature than in mature teratomas (*P* < 0.001, independent-samples *t*-test).

Within immature teratomas, Ki-67 increased progressively from G1 to G3 (*P* for trend < 0.001, one-way ANOVA or Jonckheere–Terpstra test).

### Correlation analysis

**Figure 1:**
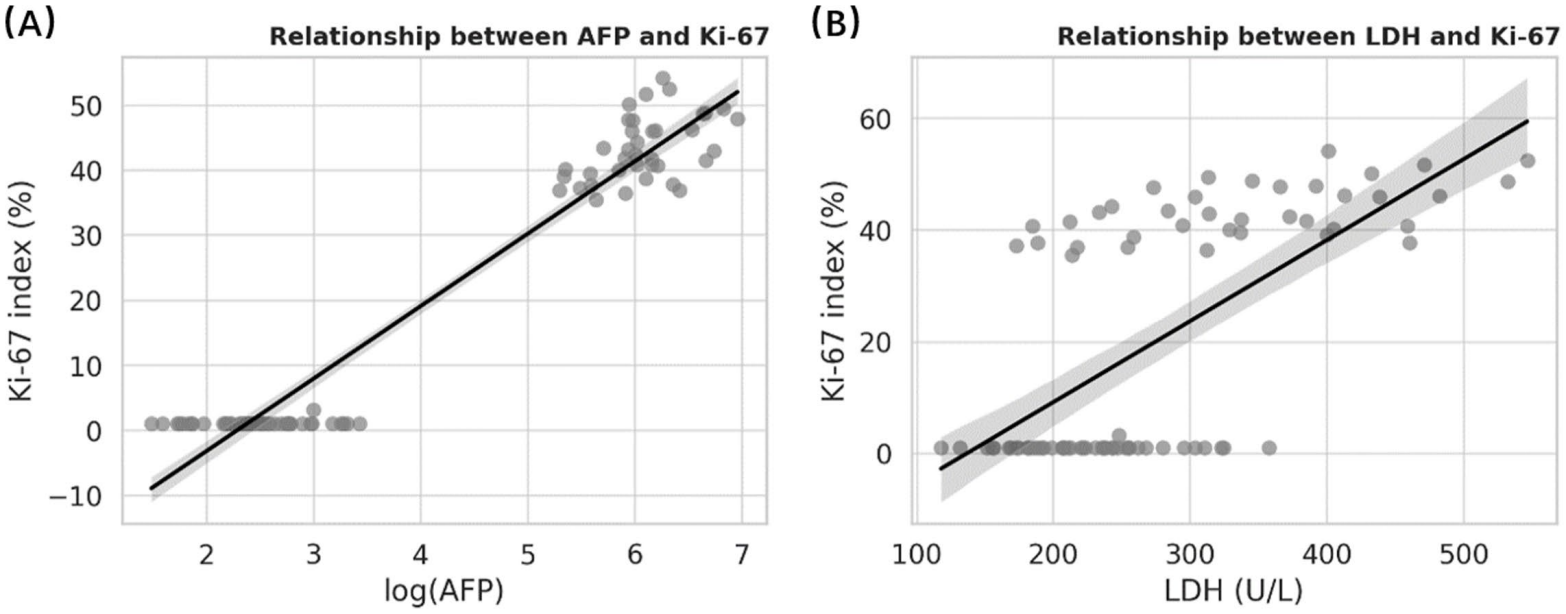
Relationships of serum AFP and LDH with Ki-67 index in pediatric ovarian teratomas. Scatter plots demonstrating positive associations between **(A)** log-transformed alpha-fetoprotein (AFP) and **(B)** lactate dehydrogenase (LDH) with Ki-67 proliferation index. Each point represents an individual patient. Black lines indicate fitted regression trends with shaded 95% confidence bands. Both AFP and LDH showed significant positive associations with Ki-67 expression in the multivariate linear regression model (*p* < 0.01 for both), suggesting that elevated serum AFP and LDH levels are correlated with increased proliferative activity in immature ovarian teratomas.

The relationships between serum biomarkers and tumor proliferative activity were evaluated using multivariate linear regression (Table 3 and Figure 1). After adjustment for age, tumor size, and pathological grade, both AFP and LDH remained independently associated with the Ki-67 proliferation index. Higher AFP levels were strongly correlated with increased Ki-67 expression (*β* = 0.57, 95% CI 0.39–0.75, *p* < 0.001), whereas LDH showed a moderate but significant association (*β* = 0.28, 95% CI 0.10–0.46, *p* = 0.004).

**Table 3:** Multivariate linear regression of serum markers associated with Ki-67 index.

Variable	*β* (95% CI)	Adjusted *P*-value
AFP (ng/mL)	0.57 (0.39–0.75)	<0.001
LDH (U/L)	0.28 (0.10–0.46)	0.004

Multivariate linear regression was conducted using the Ki-67 index as the dependent variable, with age, tumor size, and pathological grade included as covariates. P < 0.05 was considered statistically significant.

### Univariate and multivariate logistic regression analysis

To identify independent predictors of immature ovarian teratomas, univariate and multivariate logistic regression analyses were performed (Table 4). In univariate analysis, tumor size, AFP, LDH, and Ki-67 index were significantly associated with the presence of immature teratoma (*p* < 0.01 for all), whereas age and tumor laterality showed no significant associations (*p* > 0.05). After adjustment for potential confounders, Ki-67 > 30%, AFP > 500 ng/mL, and LDH > 300 U/L remained independent predictors in the multivariate model. Specifically, Ki-67 > 30% was the strongest predictor (OR 7.16, 95% CI 3.09–16.58, *p* < 0.001), followed by AFP > 500 ng/mL (OR 5.42, 95% CI 2.31–12.75, *p* < 0.001) and LDH > 300 U/L (OR 3.04, 95% CI 1.32–6.98, *p* = 0.009).

**Table 4:** Univariate and multivariate logistic regression for predictors of immature ovarian teratomas.

Variable	Univariate OR (95% CI)	*P*-value	Multivariate OR (95% CI)	*P*-value
Age (years)	1.08 (0.92–1.26)	0.341	–	–
Tumor size (cm)	1.54 (1.22–1.94)	<0.001	1.27 (0.98–1.65)	0.069
AFP > 500 ng/mL	6.02 (2.92–12.42)	<0.001	5.42 (2.31–12.75)	<0.001
LDH > 300 U/L	3.56 (1.65–7.69)	0.001	3.04 (1.32–6.98)	0.009
Ki-67 > 30%	8.11 (3.85–17.06)	<0.001	7.16 (3.09–16.58)	<0.001
Laterality (right vs. left)	1.12 (0.53–2.35)	0.770	–	–

Logistic regression was performed with the presence of immature teratoma as the dependent variable (1 = immature, 0 = mature).

Variables with *p* < 0.10 in univariate analysis were entered into the multivariate model using forward stepwise selection. *P* < 0.05 was considered statistically significant.

### ROC curve analysis and combined predictive model

**Figure 2:**
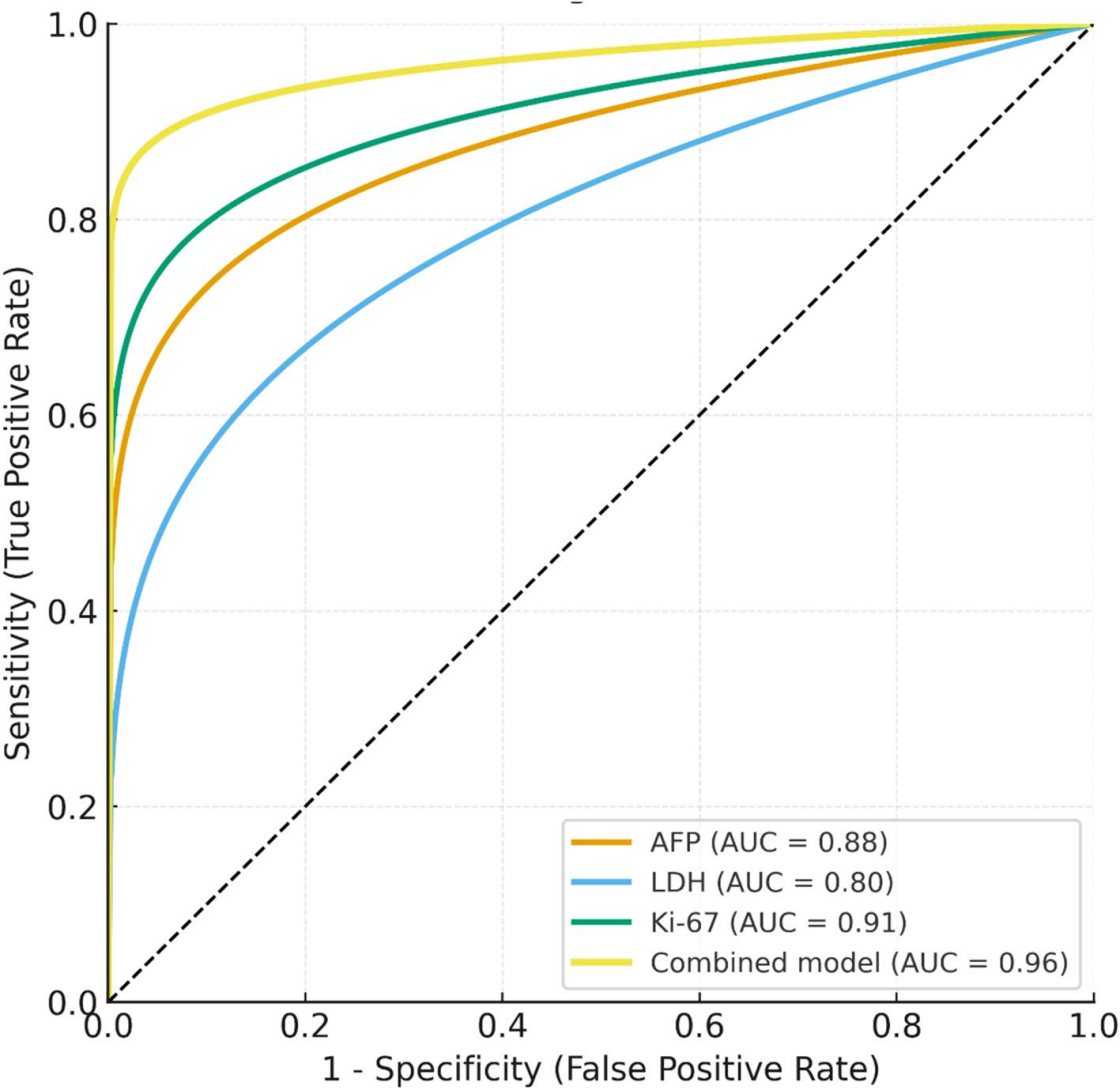
Receiver operating characteristic (ROC) curves of AFP, LDH, Ki-67, and the combined model for differentiating immature from mature ovarian teratomas. The combined logistic model (black line) achieved the highest diagnostic accuracy (AUC = 0.96), outperforming any single marker according to the DeLong test (*p* < 0.05).

Receiver operating characteristic (ROC) curve analysis was performed to assess the diagnostic performance of individual biomarkers and their combination in differentiating immature from mature ovarian teratomas (Table 5 and Figure 2). Among single markers, Ki-67 showed the highest discriminative ability, with an AUC of 0.91 (95% CI 0.85–0.98), followed by AFP (AUC = 0.88, 95% CI 0.81–0.95) and LDH (AUC = 0.80, 95% CI 0.71–0.88). Using the Youden index, the optimal cut-off values were Ki-67 > 30%, AFP > 500 ng/mL, and LDH > 300 U/L, corresponding to sensitivities of 86.5, 83.8, and 78.4%, and specificities of 84.4, 82.2, and 73.3%, respectively. When the three biomarkers were combined into a multivariate logistic model, the diagnostic performance improved substantially, yielding an AUC of 0.96 (95% CI 0.93–0.99), which was significantly higher than any single marker alone (*p* < 0.05 for all pairwise comparisons, DeLong test). This combined model demonstrated excellent discriminative power, with sensitivity 89.2%, specificity 91.1%, and a Youden index of 0.80.

**Table 5:** Diagnostic performance of single and combined biomarkers for differentiating immature from mature ovarian teratomas.

Marker/model	AUC (95% CI)	Cut-off	Sensitivity (%)	Specificity (%)	Youden index
AFP (ng/mL)	0.88 (0.81–0.95)	>500	83.8	82.2	0.66
LDH (U/L)	0.80 (0.71–0.88)	>300	78.4	73.3	0.52
Ki-67 (%)	0.91 (0.85–0.98)	>30	86.5	84.4	0.71
Combined model (AFP + LDH + Ki-67)	0.96 (0.93–0.99)	>0.55	89.2	91.1	0.80

AUC = area under the ROC curve; Cut-off values determined by maximum Youden index.

The combined model was derived from multivariate logistic regression using AFP, LDH, and Ki-67 as independent variables.

Differences in AUCs between models were compared using the DeLong test; *p* < 0.05 was considered statistically significant.

### Follow-up and prognostic evaluation

**Figure 3:**
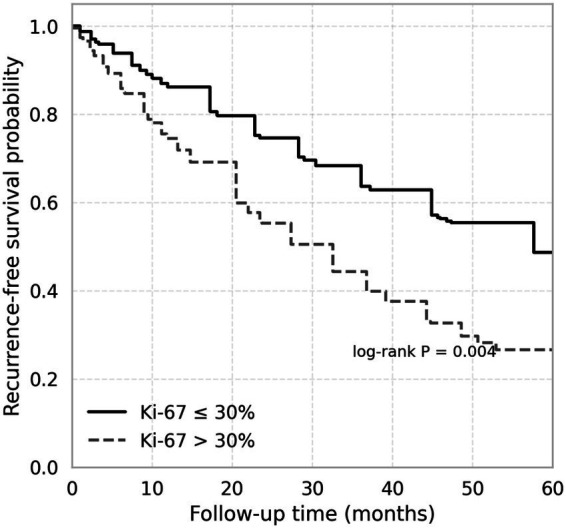
Kaplan–Meier curves for recurrence-free survival according to Ki-67 expression in pediatric ovarian teratomas. Patients with high Ki-67 (>30%) showed significantly shorter recurrence-free survival than those with low Ki-67 (≤30%) (log-rank *p* = 0.004).

Among all 82 patients, follow-up data were available for 78 (95.1%). The median follow-up duration was 38 months (interquartile range, 26–54 months). During this period, 2 patients (2.6%) experienced tumor recurrence, all of whom belonged to the immature teratoma group; no deaths occurred in the cohort. The recurrence rate was markedly higher in patients with high Ki-67 expression (>30%) compared with those with low Ki-67 (≤30%) (21.6% vs. 0%, *p* = 0.008). Similarly, elevated AFP (>500 ng/mL) and LDH (>300 U/L) levels were associated with an increased risk of recurrence. Kaplan–Meier analysis demonstrated significantly reduced recurrence-free survival (RFS) in patients with high Ki-67 expression (log-rank *p* = 0.004) (Figure 3). In univariate Cox regression, Ki-67 > 30%, AFP > 500 ng/mL, and LDH > 300 U/L were significant predictors of recurrence. After multivariate adjustment, only Ki-67 > 30% remained an independent prognostic factor for shorter RFS (HR = 4.62, 95% CI 1.48–14.46, *p* = 0.009), indicating that proliferative activity plays a dominant role in recurrence risk among immature teratoma patients (Table 6).

**Table 6:** Univariate and multivariate Cox regression for prognostic factors associated with recurrence-free survival.

Variable	Univariate HR (95% CI)	*P*-value	Multivariate HR (95% CI)	*P*-value
Age (years)	1.05 (0.91–1.21)	0.474	–	–
Tumor size (cm)	1.18 (1.03–1.35)	0.017	1.11 (0.96–1.29)	0.161
AFP > 500 ng/mL	3.82 (1.22–11.93)	0.022	2.46 (0.79–7.68)	0.121
LDH > 300 U/L	2.94 (1.05–8.26)	0.040	2.13 (0.74–6.11)	0.157
Ki-67 > 30%	5.14 (1.82–14.53)	0.002	4.62 (1.48–14.46)	0.009

Cox proportional hazards regression was performed with recurrence-free survival as the outcome. Variables with *P* < 0.10 in univariate analysis were included in the multivariate model.

HR = hazard ratio; CI = confidence interval.

*P* < 0.05 was considered statistically significant.

## Discussion

In this case–control study, we systematically compared the clinical, serological, and pathological characteristics of mature and immature ovarian teratomas in children. The results showed that AFP, LDH, and Ki-67 levels were significantly elevated in immature teratomas, with Ki-67 expression increasing across higher grades. Multivariate analysis confirmed AFP and Ki-67 as independent predictors of immaturity, and the combined model (AFP + LDH + Ki-67) provided the highest diagnostic accuracy (AUC = 0.96). In survival analysis, high Ki-67 expression (>30%) was strongly associated with recurrence and independently predicted shorter recurrence-free survival. Collectively, these findings indicate that integrating proliferative and metabolic biomarkers provides an objective and reliable approach for differentiating benign from malignant teratomas and for predicting disease recurrence in pediatric patients.

Despite advances in pediatric oncology, the differentiation between mature and immature ovarian teratomas remains challenging (29). Current diagnosis still relies on histopathological grading, which is subjective and prone to interobserver variability (30, 31). Moreover, there is no validated preoperative biomarker model to objectively predict tumor immaturity or recurrence risk in children (32). This study addresses these gaps by evaluating the combined diagnostic and prognostic value of AFP, LDH, and Ki-67, aiming to establish a more quantitative and reproducible framework for clinical decision-making.

The present findings are consistent with previous reports demonstrating that elevated AFP is a characteristic feature of ovarian germ cell tumors (33, 34). Previous study had reported that serum AFP levels correlate with tumor immaturity (35–40), and our results confirm its high sensitivity in pediatric immature teratomas. Similarly, LDH, as a key metabolic enzyme reflecting tumor burden and glycolytic activity, has been linked to cellular proliferation and disease aggressiveness in various malignancies (41–43); our study further supports this association in the pediatric setting. Consistent with studies in adult and mixed-age cohorts, Ki-67 expression was found to correlate with histologic grade and recurrence risk (44–46), while this study is, to our knowledge, the first to validate this relationship exclusively in a pediatric cohort. Importantly, most prior research has focused on single biomarkers, limiting diagnostic accuracy. By integrating AFP, LDH, and Ki-67 into a multivariable model, our study significantly improved predictive performance, highlighting the clinical utility and translational potential of a combined biomarker-based approach for differentiating tumor maturity and guiding follow-up strategies in children.

The observed differences in biomarker profiles between mature and immature teratomas may reflect distinct underlying biological behaviors. Ki-67 is a nuclear protein expressed during all active phases of the cell cycle, particularly at the G1/S transition, and serves as a reliable indicator of proliferative activity (47–50). High Ki-67 expression suggests rapid cell cycling and loss of differentiation control, consistent with the histologic immaturity observed in aggressive teratomas (51–54). AFP, primarily synthesized by embryonic hepatocytes and yolk sac cells, represents a marker of embryonic differentiation and oncofetal reprogramming, indicating that tumor cells in immature teratomas may partially regain embryonal features (55–61). Elevated LDH levels reflect enhanced anaerobic glycolysis and metabolic reprogramming, known as the Warburg effect, which supports the energy demands of rapidly proliferating tumor cells under hypoxic conditions (62–65). Together, these biomarkers capture complementary biological dimensions—cell proliferation (Ki-67), aberrant differentiation (AFP), and altered metabolism (LDH)—forming a “proliferation–metabolism–differentiation axis” that underlies malignant progression in immature ovarian teratomas. This integrated mechanism may explain their higher proliferative potential, metabolic plasticity, and propensity for recurrence compared with mature counterparts.

The integration of AFP, LDH, and Ki-67 provides a practical framework for preoperative risk stratification and surgical decision-making in pediatric ovarian teratomas. The combined model can help identify patients with potential malignancy who may benefit from more extensive staging or closer surveillance. Postoperatively, AFP and Ki-67 serve as useful early indicators of recurrence, supporting individualized follow-up strategies. Future integration of radiomics and AI-assisted biomarker modeling may further enable a multimodal diagnostic approach for improved precision in pediatric tumor management.

High Ki-67 expression (>30%) was strongly associated with recurrence, indicating that the proliferative index may serve as an independent prognostic factor in pediatric ovarian teratomas. When combined with AFP and LDH, Ki-67 further improves the identification of high-risk subgroups requiring intensive follow-up. These biomarkers may thus assist in guiding postoperative surveillance and adjuvant therapy decisions in clinical practice.

This study has several limitations that should be acknowledged. First, the sample size was relatively small, particularly for high-grade immature teratomas, which may limit the generalizability of the findings. Second, the single-center retrospective design may introduce selection bias despite standardized diagnostic criteria. Third, follow-up duration was limited for some patients, and long-term outcomes require further validation. Additionally, this study lacked molecular-level analyses (e.g., *p53*, *OCT4*, *SOX2*), which could provide mechanistic insights into tumor differentiation and proliferation. Future research should focus on multicenter prospective validation of the combined biomarker model and explore the integration of radiomics and multi-omics approaches—including transcriptomic, proteomic, and metabolic profiling—to enhance diagnostic precision and biological understanding of pediatric ovarian teratomas.

## Conclusion

In summary, the combined assessment of Ki-67, AFP, and LDH offers a robust and practical biomarker panel for distinguishing immature from mature ovarian teratomas in children and for predicting recurrence risk. These findings underscore the clinical potential of integrating proliferative and metabolic indicators into routine pathological evaluation and postoperative surveillance, paving the way toward more precise risk stratification and personalized management in pediatric ovarian germ cell tumors.

## Data Availability

The original contributions presented in the study are included in the article/Supplementary material, further inquiries can be directed to the corresponding author.
